# The Type 3 Adenylyl Cyclase Is Required for the Survival and Maturation of Newly Generated Granule Cells in the Olfactory Bulb

**DOI:** 10.1371/journal.pone.0122057

**Published:** 2015-03-25

**Authors:** Jie Luo, Xuanmao Chen, Yung-Wei Pan, Song Lu, Zhengui Xia, Daniel R. Storm

**Affiliations:** 1 Department of Pharmacology, University of Washington, Seattle, Washington, United States of America; 2 College of Life Sciences, Wuhan University, Wuhan, Hubei, China; 3 Toxicology Program in the Department of Environmental and Occupational Health Sciences, University of Washington, Seattle, Washington, United States of America; Monell Chemical Senses Center, UNITED STATES

## Abstract

The type 3 adenylyl cyclase (AC3) is localized to olfactory cilia in the main olfactory epithelium (MOE) and primary cilia in the adult mouse brain. Although AC3 has been strongly implicated in odor perception and olfactory sensory neuron (OSN) targeting, its role in granule cells (GCs), the most abundant interneurons in the main olfactory bulb (MOB), remains largely unknown. Here, we report that the deletion of AC3 leads to a significant reduction in the size of the MOB as well as the level of adult neurogenesis. The cell proliferation and cell cycle in the subventricular zone (SVZ), however, are not suppressed in AC3−/− mice. Furthermore, AC3 deletion elevates the apoptosis of GCs and disrupts the maturation of newly formed GCs. Collectively, our results identify a fundamental role for AC3 in the development of adult-born GCs in the MOB.

## Introduction

The type 3 adenylyl cyclase (AC3) is a membrane-associated, cyclic adenosine monophosphate (cAMP)-producing enzyme expressed in a wide variety of tissues [1, 2], including olfactory cilia in the main olfactory epithelium (MOE) [3–5]. It is an essential component of the olfactory signal transduction pathway [6, 7] and obligatory for MOE-mediated detection of odorants and pheromones [4, 8–10]. AC3 is also required for proper axonal projections of olfactory sensory neurons (OSNs) into the main olfactory bulb (MOB) [11, 12].

Granule cells (GCs) are the predominant inhibitory interneurons in the MOB that actively participate in modulating sensory information relayed from the OSNs [13–15]. These cells arise during embryogenesis and are persistently generated by the subventricular zone (SVZ) of the lateral ventricles (LV) throughout adulthood [16–19]. Nearly half of the adult-born cells fail to survive beyond the initial critical period [20, 21]. Remaining GCs exhibit elaborate apical dendrites in the external plexiform layer (EPL) [20, 22, 23], establish reciprocal dendrodendritic synapses with principal neurons [14, 24], and are functionally integrated into the preexisting neural circuitry of the MOB [25–27].

Several factors including sensory input [28–31], noradrenergic and cholinergic transmissions [32–34], cAMP response element-binding protein (CREB)-mediated transcription [35], as well as odorant-induced mitogen-activated protein kinase (MAPK) activation [36] are critical for the survival of newly formed GCs. However, whether odor-evoked cAMP signaling is responsible for cell survival is still open for discussion. Previous studies using anosmic mice with a mutation in cyclic nucleotide-gated channel (CNG) suggest a positive correlation between olfactory signal transduction and neuronal survival in the MOB [20, 37, 38]. Interestingly, AC3^−/−^ mice are also anosmic with impaired afferent innervation from the MOE [4, 11, 12]. Therefore, AC3-mediated cAMP signaling may contribute to the survival of newborn GCs in the MOB.

AC3 is localized to primary cilia in the MOB of adult mouse brains [39]. In addition, primary cilia are implicated in dendritic outgrowth of neocortical neurons and adult-born hippocampal neurons [40, 41]. These findings suggest the intriguing hypothesis that ciliary AC3 may regulate the maturation of newly formed GCs in the MOB.

Here, we compared the survival and maturation of newly generated GCs in the MOB of AC3^+/+^ and AC3^−/−^ mice. We discovered that the deletion of AC3 affects the size of the MOB as well as the survival and maturation of adult-born GCs. We conclude that AC3 and cAMP signaling are required for the development of new GCs in the MOB.

## Materials and Methods

### Ethics Statement

All experimental procedures were performed under protocols 2011-21 and 3041-04 approved by the Institutional Animal Care and Use Committee of the University of Washington and conformed to National Institutes of Health guidelines.

### Mice

Adult (3–6 months of age) female AC3^+/+^ and littermate AC3^−/−^ mice were bred from heterozygotes and genotyped as previously described [4]. Animals were housed in a 12 h light/dark cycle and had access to food and water *ad libitum*. A total number of 30 AC3^+/+^ mice and 24 AC3^−/−^ mice were used in the study.

### Adult neural precursor cell culture

Primary cell cultures were prepared as described [42]. Briefly, adult AC3^+/+^ mice were cervical dislocated, the SVZ were micro-dissected and enzymatically digested with 0.125% trypsin-EDTA at 37°C for 7 min followed by incubation with an equal volume of 0.014% trypsin inhibitor (Invitrogen). Tissue samples were then spun down and resuspended in serum-free culture media consisting of DMEM/F12 (Invitrogen), 1× N2 supplement (Invitrogen), 1× B27 supplement without retinoic acid (Invitrogen), 100 U/mL penicillin/streptomycin (Invitrogen), 2 mM L-glutamine (Invitrogen), 2 μg/mL heparin (Sigma), 20 ng/mL EGF (EMD Chemicals), and 10 ng/mL bFGF (Millipore). Tissues were mechanically triturated and filtered through a 40-μm cell sieve, plated in petri dishes, and cultured for 7–14 d until primary neurospheres formed. EGF and bFGF were replenished every 3 d during this period. Spheres collected from secondary passage were dissociated and plated as a monolayer culture on fibronectin and poly-L-ornithine (BD Biosciences)-coated culture plates or aclar coverslips (Electron Microscopy Sciences) for experiments.

### Immunocytochemistry

Cells were fixed with 4% paraformaldehyde and 4% sucrose in PBS at room temperature for 30 min. Fixed cells were then permeabilized with 1% SDS for 5 min, blocked in 5% bovine serum albumin (Sigma) in 0.1% Triton X-100 (Sigma) in PBS (PBST) for 2 h, and incubated with rabbit anti-AC3 (1:400; Santa Cruz Biotechnology) and TUJ-1 (1:1000; Promega) at 4°C overnight. After washing 3 times in PBST, cells were incubated with appropriate Alexa Fluor dye-conjugated secondary antibodies (Invitrogen) for 2 h. Cells were finally counterstained with 4',6-Diamidino-2-Phenylindole, Dihydrochloride (DAPI, 2 μg/mL; Invitrogen) and mounted onto slides using Aqua-Poly/Mount (Polysciences).

### Bromodeoxyuridine (BrdU) injections

BrdU (Sigma) was dissolved in sterile saline and filtered at 0.22 μm before use. To examine cell proliferation in the SVZ, mice were given a single dose of BrdU at 100 mg/kg body weight by intraperitoneal injection and perfused 2 h later. To examine adult neurogenesis in the MOB, mice were given intraperitoneal injections of BrdU at 100 mg/kg body weight 3 times per day (every 2 h for 6 h) for 3 consecutive days and perfused 28 d later.

### Adeno-associated virus serotype 1 expressing green fluorescent protein (AAV1-GFP) microinjections

AAV1-GFP was purchased from Penn Vector Core and the viral titer was 10^13^ colony-forming units/mL. Mice were anesthetized with a ketamine/xylazine mixture and aligned in a stereotaxic apparatus. 0.2 μL virus was delivered at 2 coordinates bilaterally (relative to bregma: 1 mm anterior, ± 1 mm lateral, 2.2 mm ventral; 0 mm anterior, ± 1.4 mm lateral, 1.6 mm ventral) via a Hamilton syringe using a microsyringe pump controller (World Precision Instruments). Mice were sacrificed at 28 d post-injection.

### Immunohistochemistry

Mice were anesthetized with an intraperitoneal injection of 20 mL/kg body weight of a mixture of ketamine (7 mg/mL) and xylazine (0.44 mg/mL). Mice were then perfused transcardially with saline followed by 4% paraformaldehyde in PBS. Brains were removed, fixed overnight in the same fixative, and transferred into 30% sucrose in PBS until sunk. After OCT (Sakura) embedding, brains were cut into serial sections (40 or 50 μm) in a cryostat (Leica). For BrdU staining, sections were pretreated in 2 N HCl at 37°C for 30 min followed by 0.1 M borate buffer (pH 8.5) for 10 min. Sections were then permeabilized with PBST for 10 min, blocked in 10% goat serum or donkey serum (Sigma) in PBST for 1 h, and incubated with the following primary antibodies at 4°C overnight: rabbit anti-AC3 (1:400; Santa Cruz Biotechnology); rat anti-BrdU (1:200; AbD Serotec); rabbit anti-cleaved caspase-3 (1:200; Cell Signaling Technology); goat anti-doublecortin (DCX, 1:200; Santa Cruz Biotechnology); mouse anti-GFP (1:500; Invitrogen); rabbit anti-Ki67 (1:200; Vector Labs), mouse anti-NeuN (1:100; Millipore); rabbit anti-NeuN(1:400; Millipore); rabbit anti-somatostatin receptor 3 (SSTR3, 1:1000; Thermo Scientific). After washing 3 times in PBST, sections were incubated with appropriate Alexa Fluor dye-conjugated secondary antibodies (Invitrogen) for 2 h. For AC3 and SSTR3 co-localization, sections were first stained with rabbit anti-SSTR3 (1:20,000; Thermo Scientific). The signal was amplified by the deposition of cyanine-3 tyramide complexes using Tyramide Signal Amplification System (PerkinElmer Life and Analytical Sciences). Sections were then blocked in 10% goat serum and incubated with rabbit anti-AC3 (1:400; Santa Cruz Biotechnology). Sections were finally counterstained with DAPI (2 μg/mL; Invitrogen) and coverslipped using Aqua-Poly/Mount (Polysciences). Secondary antibodies alone were used as negative controls.

### Histology

Sections were washed 3 times in PBS followed by ddH_2_O before being mounted onto the slides (VWR). Sections were stained in 0.1% cresyl violet (Sigma) for 20 min with intermittent rocking. Sections were then dehydrated through an ascending gradient of ethanol (70% EtOH, 90% EtOH, and 100% EtOH), cleared by two changes of xylene, and coverslipped with Permount (Fisher Scientific).

### Imaging and analysis

Sections were imaged on either a Zeiss AxioImager M2 microscope or a Zeiss 510 Meta laser scanning confocal microscope. Images were uniformly processed for contrast and brightness using Adobe Photoshop CS for experimental comparisons. To quantify the numbers of BrdU^+^, caspase 3^+^, Ki67^+^ and NeuN^+^ cells, 40-μm-thick coronal sections spanning the entire MOB or the anterior SVZ (0–1.18 mm anterior relative to bregma) were examined in the study. To assess the total dendritic length and dendritic branching number of newly generated GFP^+^ cells, 50-μm-thick sagittal sections were analyzed using Simple Neurite Tracer plug-in of Fiji software (National Institutes of Health).

### Size measurement of brain regions

Volume estimation of the MOB and each sublayer was conducted using the Cavalieri Estimator probe of Stereo Investigator software (MBF Bioscience). A 100-μm point grid was overlaid unbiasedly onto every sixth section stained with cresyl violet.

### Quantification of immunostained cells

The density of BrdU^+^ and NeuN^+^ cells in the granule cell layer (GCL) of the MOB and the density of BrdU^+^ and Ki67^+^ cells in the SVZ were quantified using the Optical Fractionator probe of Stereo Investigator software (MBF Bioscience). Specifically, we performed systematic sampling of BrdU^+^ cells in the SVZ in every third section with the grid set at 100 × 50 μm^2^ and the counting frame set at 25 × 25 μm^2^. For cells in the MOB, a 350-μm grid was overlaid unbiasedly onto every sixth section with the counting frame set at 100×100 μm^2^. Because caspase 3^+^ cells were rarely encountered, sampling of these cells was done exhaustively throughout the GCL. The total number of immunopositive cells was then divided by the total volume of the GCL to give an estimate of the density of caspase 3^+^ cells.

### Statistical analysis

Results are expressed as mean ± SEM. Data were analyzed by unpaired two-tailed Student’s *t* test. Significance was set at *p* < 0.05.

## Results

### AC3 is predominately expressed by primary cilia of GCs in the MOB

**Fig 1:**
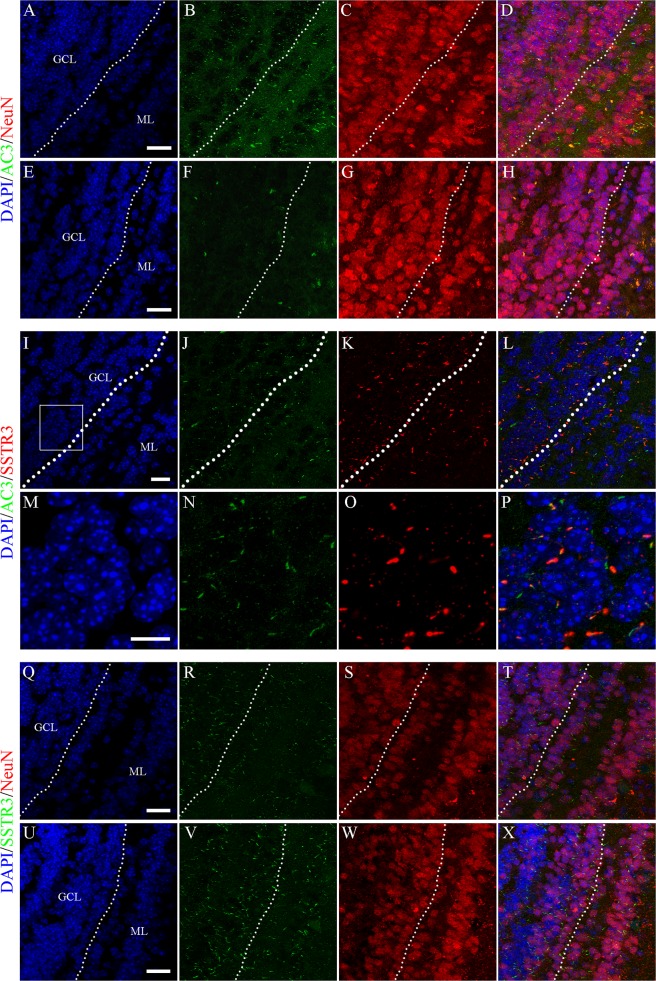
AC3 is highly concentrated on primary cilia of GCs in the MOB. (A-H) Representative images of AC3 (green) and NeuN (red) staining in the GCL of AC3^+/+^ (A-D) and AC3^−/−^ mice (E-H). Nuclei were counterstained with DAPI (blue). Scale bar, 20 μm. GCL, granule cell layer; ML, mitral cell layer. Dashed lines indicate GCL contour. (I-L) Representative images of AC3 (green) and SSTR3 (red) staining in the GCL of AC3^+/+^ mice. Nuclei were counterstained with DAPI (blue). Scale bar, 20 μm. GCL, granule cell layer; ML, mitral cell layer. Dashed lines indicate GCL contour. (M-P) Higher magnification of the boxed area in (I). Scale bar, 10 μm. (Q-X) Representative images of SSTR3 (green) and NeuN (red) staining in the GCL of AC3^+/+^ (Q-T) and AC3^−/−^ mice (U-X). Nuclei were counterstained with DAPI (blue). Scale bar, 20 μm. GCL, granule cell layer; ML, mitral cell layer. Dashed lines indicate GCL contour.

AC3-positive cilia have been detected throughout the MOB in the adult mouse brain [39]. To determine whether AC3 is highly expressed by primary cilia of GCs in the MOB, we immunostained sections from AC3^+/+^ mice with antibodies against AC3 and NeuN, a mature neuron marker [43]. AC3-decorated primary cilia were observed protruding out of virtually all NeuN^+^ cells in the GCL (Fig. 1A-D). In sharp contrast, AC3 expression was completely absent in AC3^−/−^ mice (Fig. 1E-H), confirming that the antibody was indeed specific. In addition, to investigate whether AC3 is indispensible for the stability of primary cilia, we analyzed the distribution of SSTR3, another prominent ciliary marker [44, 45], in neurons within the GCL. Strong co-localization of AC3 and SSTR3 in primary cilia of GCs were detected on OB sections of AC3^+/+^ mice (Fig. 1I-P), suggesting that AC3 is present in cilia and not other small tubular structures. In addition, rod-shaped SSTR3-immunoreactive structures were discernible in both AC3^+/+^ (Fig. 1Q-T) and AC3^−/−^ mice (Fig. 1U-X). These results suggest that AC3 is selectively targeted to primary cilia of GCs in the MOB but not required for cilia structure.

### AC3 deletion reduces MOB size

**Fig 2:**
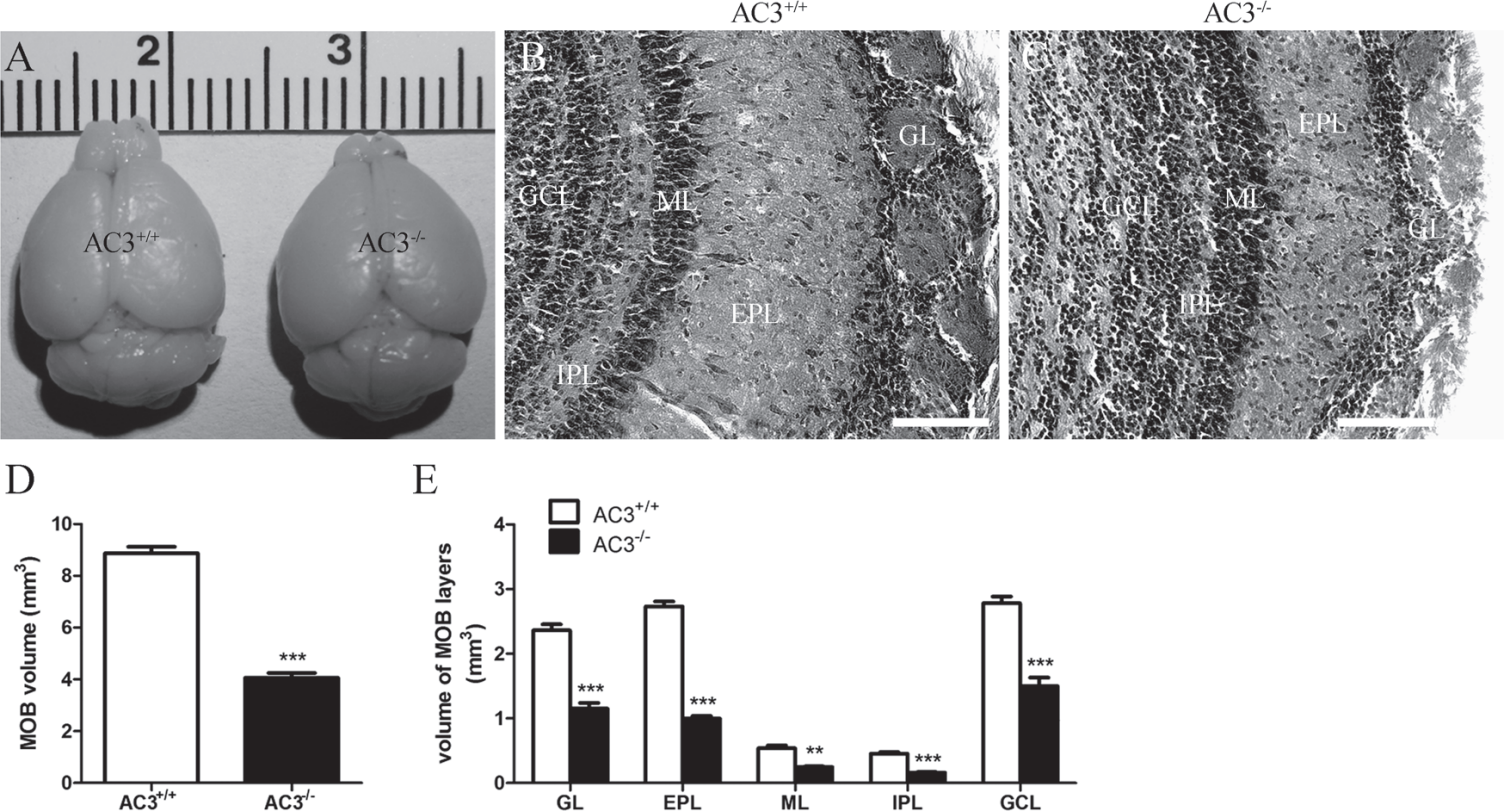
AC3^−/−^ mice demonstrate a much smaller MOB. (A) Post-fixed brains from AC3^+/+^ and AC3^−/−^ mice. (B-C) Nissl-stained coronal sections from AC3^+/+^ (B) and AC3^−/−^ mice (C). Scale bar, 100 μm. GL, glomerular layer; EPL, external plexiform layer; ML, mitral cell layer; IPL, internal plexiform layer; GCL, granule cell layer. (D) Stereological measurements of the MOB volume of AC3^+/+^ and AC3^−/−^ mice. n = 3–4 per genotype. ***p < 0.001; unpaired Student’s *t* test. (E) Stereological measurements of the volume of MOB sublayers of AC3^+/+^ and AC3^−/−^ mice. n = 3–4 per genotype. **p < 0.01, ***p < 0.001, unpaired Student’s *t* test.

We previously demonstrated that AC3^−/−^ mice are anosmic and exhibit a complete loss of electro-olfactogram responses in the MOE to olfactory cues [4, 8–10]. Interestingly, disruption of olfactory activity is often associated with a remarkable reduction in OB size [20, 37, 38, 46, 47]. To evaluate whether the overall structure of the MOB is affected by AC3 deletion, we performed unbiased stereological examination on Nissl-stained OB series. The OBs in AC3^−/−^ mice were much smaller than those in AC3^+/+^ controls (Fig. 2A). The laminar organization, however, was still distinguishable in AC3^−/−^ animals (Fig. 2B-C). Further measurements revealed a significant decrease of 45.8% in the size of the MOB of AC3^−/−^ mice compared with AC3^+/+^ controls (Fig. 2D; AC3^+/+^: 8.868 ± 0.2508 mm^3^, n = 4; AC3^−/−^: 4.061 ± 0.1829 mm^3^, n = 3; *t* test, *p* < 0.0001). Moreover, the size of each sub-layer was also substantially compromised in AC3^−/−^ mice (Fig. 2E; GL (glomerular layer), *t* test, *p* = 0.0003; EPL, *t* test, *p* < 0.0001; ML (mitral cell layer), *t* test, *p* = 0.0019; IPL (internal plexiform layer), *t* test, *p* = 0.0003; GCL, *t* test, *p* = 0.0005). These data suggest that the deletion of AC3 results in a profound reduction in the volume of the MOB.

### AC3 deletion reduces the number of adult-born GCs in the MOB without inhibiting cell proliferation in the SVZ

**Fig 3:**
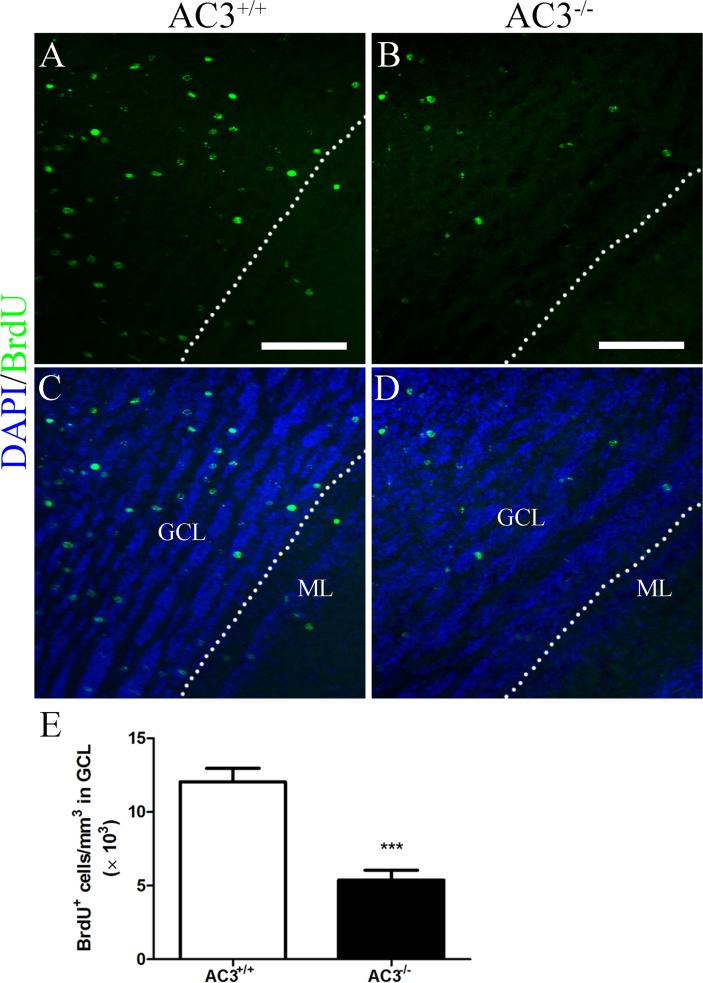
Adult neurogenesis in the MOB is reduced in AC3^−/−^ mice. (A-D) Representative images of BrdU (green) staining in the GCL of AC3^+/+^ (A, C) and AC3^−/−^ mice (B, D) at 28 d post-BrdU injection. Nuclei were counterstained with DAPI (blue). Scale bar, 100 μm. GCL, granule cell layer; ML, mitral cell layer. Dashed lines indicate GCL contour. (E) Quantification of BrdU^+^ cells in the GCL of AC3^+/+^ and AC3^−/−^ mice at 28 d post-BrdU injection. n = 5 per genotype. ***p < 0.001, unpaired Student’s *t* test.

Olfactory sensory input plays a pivotal role in promoting the survival of newly generated neurons in the MOB [28–31, 48, 49]. To examine the function of AC3 in adult neurogenesis, we injected animals with BrdU 3 times per day for 3 consecutive days, and quantified the total number of label-retaining cells in the MOB 28 d after the last BrdU injection. The vast majority of BrdU-labeled cells were detected within the GCL of the MOB in both AC3^+/+^ and AC3^−/−^ mice (Fig. 3A-D). However, the number of BrdU^+^ cells in AC3^−/−^ mice was reduced by more than half relative to AC3^+/+^ controls (Fig. 3E; AC3^+/+^: 12.04 ± 0.9103 × 10^3^/mm^3^, n = 5; AC3^−/−^: 5.382 ± 0.6612 × 10^3^/mm^3^, n = 5; *t* test, *p* = 0.0004). These data suggest that the deletion of AC3 decreases the number of adult-born GCs in the MOB.

**Fig 4:**
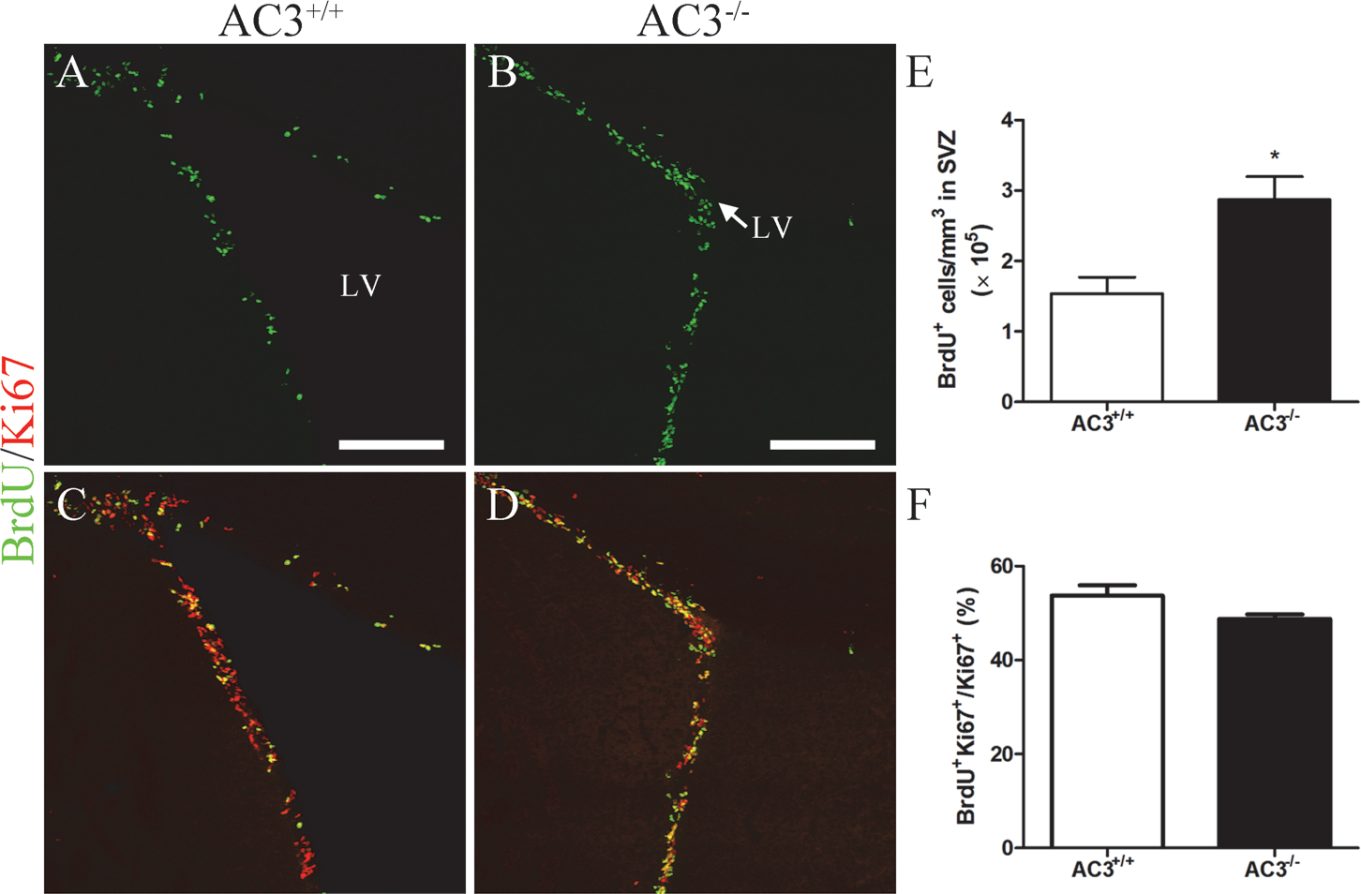
Neither cell proliferation nor cell cycle in the SVZ is attenuated in AC3^−/−^ mice. (A-D) Representative images of BrdU (green) and Ki67 (red) staining in the SVZ of AC3^+/+^ (A, C) and AC3^−/−^ mice (B, D) at 2 h post-BrdU injection. Scale bar, 200 μm. LV, lateral ventricle. The arrow in (B) indicates a closed ventricle. (E) Quantification of BrdU^+^ cells in the SVZ of AC3^+/+^ and AC3^−/−^ mice at 2 h post-BrdU injection. n = 4 per genotype. *p < 0.05, unpaired Student’s *t* test. (F) The percentage of Ki67^+^ cells also labeled with BrdU in the SVZ of AC3^+/+^ and AC3^−/−^ mice at 2 h post-BrdU injection. n = 4 per genotype.

The newly formed GCs in the MOB are persistently generated by the SVZ of adult mammalian brains [50–52]. To assess whether attenuated adult neurogenesis in AC3^−/−^ mice results from alterations in SVZ proliferation, we injected animals with a single dose of BrdU and quantified the total number of label-retaining cells along the SVZ 2 h post-injection. Neuroblasts in the SVZ exhibited AC3^+^ primary cilia (S1 Fig.). Surprisingly, the number of BrdU^+^ cells in AC3^−/−^ mice was actually increased almost 2-fold relative to AC3^+/+^ controls (Fig. 4A, B and E; AC3^+/+^: 1.540 ± 0.2322 × 10^5^/mm^3^, n = 4; AC3^−/−^: 2.873 ± 0.3263 × 10^5^/mm^3^, n = 4; *t* test, *p* = 0.0159). We also analyzed the ratio of dividing cells (BrdU^+^Ki67^+^) over total number of cycling cells (Ki67^+^) to estimate cell cycle length in the SVZ. No significant difference was detected in the percentage of Ki67^+^ cells that were co-labeled with BrdU between AC3^−/−^ mice and AC3^+/+^ controls (Fig. 4C, D and F; AC3^+/+^: 53.72 ± 2.189%, n = 4; AC3^−/−^: 48.77 ± 0.9701%, n = 4; *t* test, *p* = 0.0840). These results suggest that the deletion of AC3 does not suppress cell proliferation or cell cycle in the SVZ. The findings that AC3^−/−^ mice exhibit a doubling of proliferating progenitors in the SVZ but only half of newly formed GCs in the MOB relative to AC3^+/+^ controls indicate that the survival and maturation of SVZ-derived cells may be severely impeded in mutant animals.

### AC3 deletion perturbs the survival of GCs in the MOB

**Fig 5:**
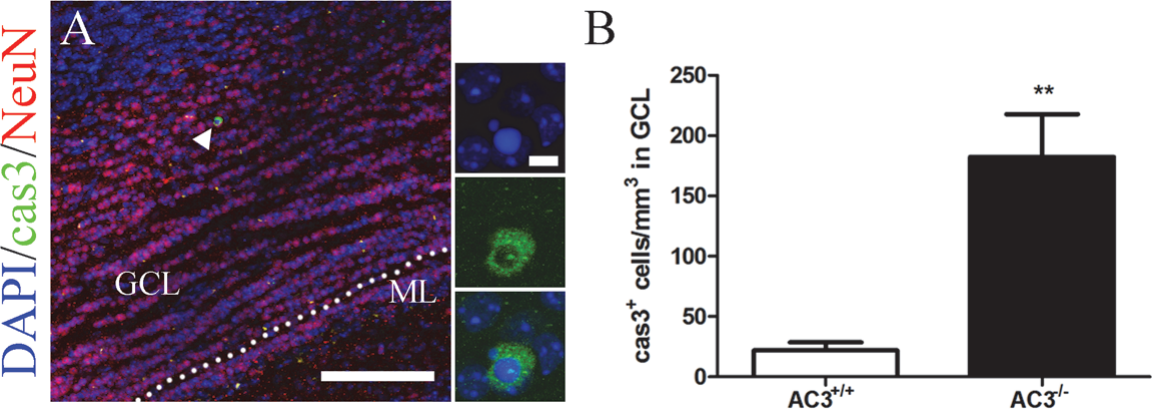
The apoptosis of GCs in the MOB is elevated in AC3^−/−^ mice. (A) Representative images of active caspase-3 (green) and NeuN (red) staining in the GCL of AC3^−/−^ mice. Nuclei were counterstained with DAPI (blue). Scale bar, 100 μm. GCL, granule cell layer; ML, mitral cell layer. Dashed lines indicate GCL contour. The apoptotic GC indicated by arrowhead in (A) is shown at higher magnification in the vertical panels on right (scale bar, 5 μm). (B) Quantification of caspase-3^+^ cells in the GCL of AC3^+/+^ and AC3^−/−^ mice. n = 5 per genotype. **p < 0.01, unpaired Student’s *t* test.

An increase in apoptotic GCs has been detected in sensory-deprived animals [20, 29, 49, 53, 54]. To monitor the level of GC death in anosmic AC3^−/−^ mice, we quantified the number of cells that were immunopositive for active caspase-3, an enzyme critically involved in the mammalian apoptotic pathway [55, 56]. Caspase-3^+^ cells in the GCL of AC3^−/−^ mice (Fig. 5A) were about 8 times greater than AC3^+/+^ controls (Fig. 5B; AC3^+/+^: 21.54 ± 6.763/mm^3^, n = 5; AC3^−/−^: 182.4 ± 35.39/mm^3^, n = 5; *t* test, *p* = 0.0021). These results indicate that the deletion of AC3 leads to a pronounced elevation in apoptotic elimination of GCs in the MOB.

### AC3 deletion impairs the maturation of newly generated GCs

**Fig 6:**
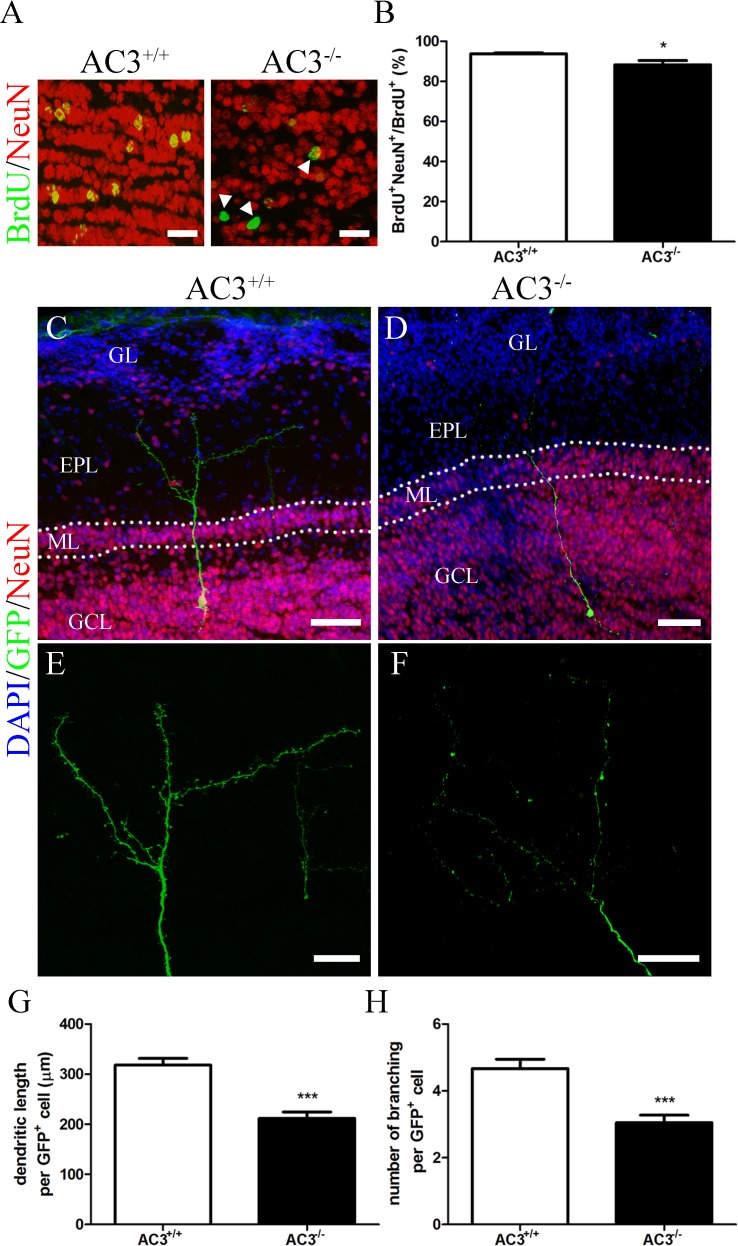
The maturation of newly generated GCs is impaired in AC3^−/−^ mice. (A) Representative images of BrdU (green) and NeuN (red) staining in the GCL of AC3^+/+^ (left) and AC3^−/−^ mice (right) at 28 d post-BrdU injection. Scale bar, 20 μm. Arrowheads indicate BrdU^+^ cells that have not become neurons (BrdU^+^/NeuN^−^). (B) The percentage of BrdU^+^ cells also labeled with NeuN in the GCL of AC3^+/+^ and AC3^−/−^ mice at 28 d post-BrdU injection. n = 5 per genotype. *p < 0.05, unpaired Student’s *t* test. (C-D) Superimposed images of GFP (green) and NeuN (red) staining in the MOB of AC3^+/+^ (C) and AC3^−/−^ mice (D) at 28 d post-virus injection. Nuclei were counterstained with DAPI (blue). Scale bar, 50 μm. GL, glomerular layer; EPL, external plexiform layer; ML, mitral cell layer; GCL, granule cell layer. Dashed lines indicate ML contour. (E-F) Enlarged dendritic arbors of the GFP^+^ cell in (C) and (D) respectively. Scale bar, 25 μm. (G) Quantification of the average dendritic length in the EPL of AC3^+/+^ (n = 3, 57 cells) and AC3^−/−^ mice (n = 3, 42 cells). ***p < 0.001, unpaired Student’s *t* test. (H) Quantification of the average number of dendritic branching in the EPL of AC3^+/+^ (n = 3, 57 cells) and AC3^−/−^ mice (n = 3, 42 cells). ***p < 0.001, unpaired Student’s *t* test.

Young neurons originated from the SVZ migrate along the rostral migratory stream (RMS) into the MOB where they further differentiate into mature neurons [52, 57–59]. cAMP has been shown to promote the differentiation of SVZ progenitors isolated from developing brains [60]. To determine whether neuronal differentiation in adult MOB also requires AC3 and cAMP signaling, we quantified the fraction of newly generated neurons by counting the number of NeuN-positive (BrdU^+^NeuN^+^) cells relative to the number of cells positive for BrdU in the GCL 28 d after the last BrdU injection. Over 90% of BrdU^+^ cells were co-immunopositive for NeuN in AC3^+/+^ mice (Fig. 6A). However, the ratio of BrdU^+^NeuN^+^ cells/BrdU^+^ cells decreased slightly in AC3^−/−^ mice (Fig. 6B; AC3^+/+^: 93.82 ± 0.3883%, n = 5; AC3^−/−^: 88.30 ± 2.050%, n = 5; *t* test, *p* = 0.0294), suggesting a minor delay in neuronal maturation.

Within the MOB, young GCs also undergo a series of morphological changes over time and finally develop elaborate, branched, apical dendrites with spines in the EPL [20, 22, 23]. To explore whether the formation of dendritic arbors in newly generated GCs depends on AC3, we injected AAV1-GFP into the SVZ and examined the morphology of GFP^+^ cells in the MOB 28 d post-injection. GFP^+^ cells of AC3^+/+^ mice extended a long apical dendrite with multiple branches in the EPL (Fig. 6C). Densely-packed spines, a typical morphology of class 5 cells [20, 22], were also visible along the dendritic arbors (Fig. 6E). However, GFP^+^ cells of AC3^−/−^ mice possessed fewer dendritic branches in the EPL and no associated spiny protrusions, corresponding to class 4 cells only (Fig. 6D and F). We also measured the dendritic length and the branching number per GFP^+^ cell as an index of neuronal maturation. AC3^−/−^ mice exhibited a 67% decrease in the dendritic length (Fig. 6G; AC3^+/+^: 318.2 ± 13.28 μm, n = 3, 57 cells; AC3^−/−^: 211.8 ± 12.62 μm, n = 3, 42 cells; *t* test, *p* < 0.0001) and a 65% decrease in the branching number (Fig. 6H; AC3^+/+^: 4.667 ± 0.2802, n = 3, 57 cells; AC3^−/−^: 3.048 ± 0.2259, n = 3, 42 cells; *t* test, *p* < 0.0001) compared with AC3^+/+^ controls. These data indicate that the deletion of AC3 blocks dendritic complexity of newborn GCs.

## Discussion

AC3 is highly enriched in olfactory cilia of the MOE in adult mice [3–5]. This G protein-coupled adenylyl cyclase mediates the detection of odorants and pheromones by the MOE through sequential activation of critical components of the olfactory signal transduction cascade [4, 6, 7]. AC3 is also detected in primary cilia of the MOB [39]. The functional significance of ciliary AC3 in the MOB, however, has not been defined yet. In this study, we investigated the importance of AC3 for the survival and maturation of GCs, the leading population of interneurons in the MOB. This was accomplished by comparing AC3^−/−^ and AC3^+/+^ mice. We discovered that AC3 is required for the size of the MOB and the level of adult neurogenesis. In addition, AC3 regulates the survival and maturation of newly formed GCs in the MOB.

### AC3 and primary cilia

Primary cilia are microtubule-based, non-motile appendages that protrude from the surface of almost all mammalian cells [61–63]. Although the functional relevance of these tiny organelles is still poorly understood, certain proteins have been detected specifically localized to primary cilia including serotonin receptor 6 (5-HT_6_), SSTR3 and AC3 [39, 44, 45, 64, 65]. Previous work has demonstrated a widespread distribution of AC3^+^ cilia throughout the MOB in the adult mouse brain [39]. Here, we observed abundant antenna-like structures expressing AC3 in all sublayers of the MOB (Fig. 1A-D). Moreover, the presence of primary cilia was revealed by SSTR3 immunoreactivity in both AC3^+/+^ and AC3^−/−^ mice (Fig. 1Q-X). These findings are in accordance with previous studies of hippocampal neurons [66], which suggest that AC3 is not imperative for the formation of primary cilia although it could be important for the length or other physical properties of primary cilia.

### AC3 and the MOB

The MOB is a laminated structure that functions as the relay station where olfactory information from the MOE is processed and integrated before being transmitted towards higher cortical regions [14, 15, 67, 68]. It evaginates from the rostral telencephalon at E12.5 [69, 70], coinciding with the time when AC3 first appears in the OSNs [11]. However, unlike fibroblast growth factor signaling [71, 72], AC3 is not essential for MOB morphogenesis. AC3 null mice still develop a MOB but with aberrant glomerular organization detected as early as P15 [10–12]. Here, we characterized gross morphological changes in the MOB of adult AC3^−/−^ mice. The loss of AC3 results in a more than 50% reduction in the size of the MOB (Fig. 2). It has been established that the volume of the MOB is closely associated with olfactory activity [20, 37, 38, 46, 47, 73]. Since adult AC3^−/−^ mice lack odor-evoked responses at both behavioral and electrophysiological levels [4, 8–10, 74], we hypothesize that the absence of sensory input may contribute to the reduced MOB in AC3^−/−^ mice.

### AC3 and GC survival

The addition of newly generated GCs to bulbar circuitry is a highly competitive process in which half of those originated from the SVZ undergo apoptotic elimination [20, 21]. Odor enrichment and olfactory learning have been suggested to enhance the survival of adult generated neurons [30, 31, 75–77], whereas sensory deprivation reduces the number of newborn cells in the MOB [20, 28, 29, 35, 48, 49]. In this study, we compared the number of newly formed GCs in the MOB between AC3^+/+^ and AC3^−/−^ mice. The number of surviving adult-born GCs is dramatically reduced in AC3^−/−^ mice (Fig. 3). This effect is accompanied by elevated caspase 3^+^ profiles in the GCL (Fig. 5). These data indicate that the attenuated neurogenesis in the MOB of AC3^−/−^ mice is most likely due to an increase in GC death.

A reduction in surviving GCs in the MOB of AC3^−/−^ mice may also result from increased proportion of newly generated neuroblasts undergo programmed cell death as they migrate into the bulb. In addition, it has been proposed that primary cilia may play a role in neuronal polarity and migration [78]. The chain and radial migration of neuroblasts, therefore, are likely to be disrupted in AC3^−/−^ mice. We could not rule out these possibilities in our study.

The substantially enhanced cell proliferation in the SVZ of AC3^−/−^ mice is noteworthy (Fig. 4). In fact, both decreased [79] and unchanged [20, 28] levels of SVZ proliferation have been reported in various anosmic models. The discrepancy of our work and that of others may attribute to the differences in the methods and the extent of sensory deprivation. More importantly, since AC3 is present in primary cilia of the SVZ stem cell niche (S1 Fig.), alterations other than olfactory deficits may also explain an increase in SVZ proliferation in our case. cAMP regulates proliferation in a cell-specific manner [80]. It has already been known that the activation of the cAMP-CREB pathway promotes cell proliferation in the dentate gyrus of the hippocampus [81, 82]. Our results, however, imply an inhibitory role of cAMP signaling in SVZ proliferation. Moreover, Bischofberger and colleagues have demonstrated that the differentiation of SVZ-derived neural progenitors into mature neurons requires cAMP signaling [60]. Furthermore, possible disruptions in neuroblasts migration as discussed above may also account for the accumulation of dividing cells in the SVZ of AC3^−/−^ mice. Nevertheless, our findings exclude the possibility that reduced neurogenesis in AC3^−/−^ mice results from attenuated expansion of neural progenitors in the SVZ.

### AC3 and GC maturation

After differentiation, newborn GCs exhibit antigenic and morphologic characteristics equivalent to mature neurons [83]. Various biochemical markers including NeuN, gamma-aminobutyric acid (GABA), calretinin and N-copine have been detected in the GCL region [84, 85]. Lineage tracing experiments have revealed that newly generated cells start to express NeuN 14 d after birth [86]. In addition, the population of BrdU^+^NeuN^+^ cells increases considerably and takes up approximately 90% of all BrdU-labeled cell by 1 month [86]. In our study, we also detected that 94% of BrdU^+^ cells co-expressed NeuN in the GCL of AC3^+/+^ controls 28 d post-BrdU injection (Fig. 6A and B). This ratio is somewhat higher than that in AC3^−/−^ mice, implying a cAMP-dependent differentiation of neuroblasts.

A striking morphological feature of fully developed GCs is the extending of elaborate, branched dendrites with dense spiny protrusions into the EPL [20, 22, 23], where they form synaptic connections with principal neurons of the MOB [14, 24]. The majority of newborn GCs differentiate into class 5 cells 30 d after birth [20]. Consistent with these findings, we also observed mature GCs in AC3^+/+^ mice 28 d after injecting AAV1-GFP virus into the SVZ (Fig. 6C and E). However, GFP^+^ cells in AC3^−/−^ mice only acquire characteristics corresponding to class 4 cells (Fig. 6D and F) and exhibit less complicated dendritic architecture (Fig. 6G-H). These results suggest that AC3 and cAMP signaling may facilitate structural maturation of newborn GCs. The involvement of cAMP-CREB cascade in dendrite elaboration has been described in adult-born hippocampal neurons and SVZ-derived cells in culture [35, 87, 88]. More interestingly, recent studies have also suggested a crucial role of primary cilia in dendritic organization [40, 41]. It is highly speculative that cAMP signals generated by AC3 in primary cilia promote morphological maturation of adult generated GCs in the MOB.

## Conclusions

In summary, we report that the survival and maturation of newly formed GCs are severely perturbed in AC3^−/−^ mice. Because AC3 is present in olfactory cilia of the MOE and primary cilia of the MOB, we conclude that both incoming activity and local cAMP signaling may be required for the development of GCs in the MOB.

## Supporting Information

S1 Fig Neuroblasts express AC3 in primary cilia. (A-D) Representative images of AC3 (green) and DCX (red) staining in the SVZ of AC3^+/+^ mice. Nuclei were counterstained with DAPI (blue). Scale bar, 25 μm. LV, lateral ventricle. (E-H) Representative images of AC3 (green) and TUJ-1 (red) staining on SVZ-derived neural precursors *in vitro*. Nuclei were counterstained with DAPI (blue). Scale bar, 10 μm. Arrowheads indicate primary cilia
(TIF)
