# Supplementary figures and images for: The Type 3 Adenylyl Cyclase Is Required for the Survival and Maturation of Newly Generated Granule Cells in the Olfactory Bulb
Source: PLoS One. 2015 Mar 25;10(3):e0122057. doi: 10.1371/journal.pone.0122057 (PMC4373939; doi:10.1371/journal.pone.0122057)

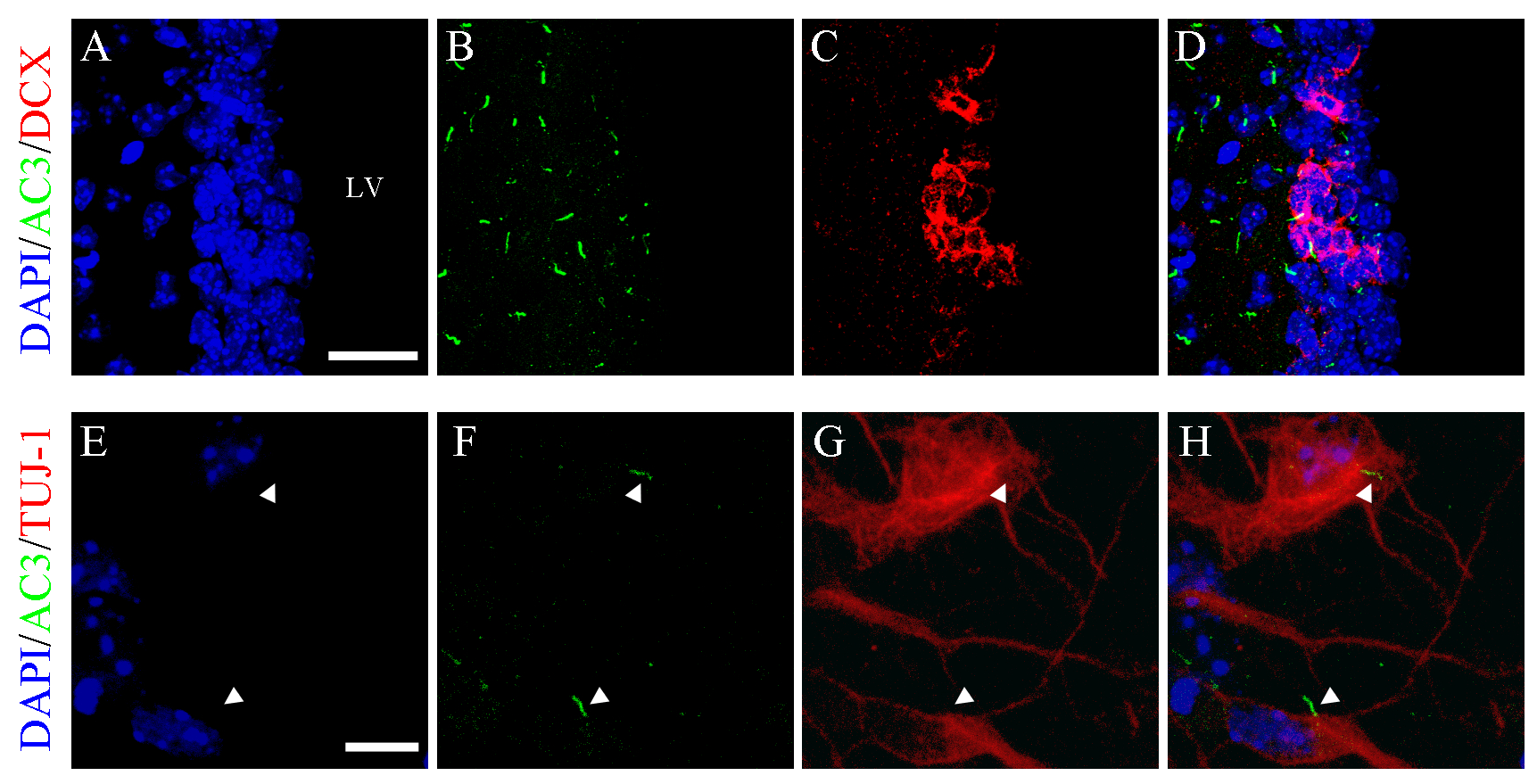

Supplement: S1 Fig — (A-D) Representative images of AC3 (green) and DCX (red) staining in the SVZ of AC3+/+ mice. Nuclei were counterstained with DAPI (blue). Scale bar, 25 μm. LV, lateral ventricle. (E-H) Representative images of AC3 (green) and TUJ-1 (red) staining on SVZ-derived neural precursors in vitro. Nuclei were counterstained with DAPI (blue). Scale bar, 10 μm. Arrowheads indicate primary cilia (TIF) [file pone.0122057.s001.tif]
